# From global change to a butterfly flapping: biophysics and behaviour affect tropical climate change impacts

**DOI:** 10.1098/rspb.2014.1264

**Published:** 2014-10-22

**Authors:** Timothy C. Bonebrake, Carol L. Boggs, Jeannie A. Stamberger, Curtis A. Deutsch, Paul R. Ehrlich

**Affiliations:** 1 Department of Earth Sciences, School of Biological Sciences, University of Hong Kong, Pokfulam Road, Hong Kong SAR, Hong Kong; 2 Department of Biological Sciences, University of South Carolina, Columbia, SC 29208, USA; 3 Disaster Resilience Leadership Academy, Tulane University, New Orleans, LA 70118, USA; 4 School of Oceanography, University of Washington, Seattle, WA 98195, USA; 5 Department of Biology, Stanford University, Stanford, CA 94305, USA

**Keywords:** climate change, biodiversity, tropics, biophysics

## Abstract

Difficulty in characterizing the relationship between climatic variability and climate change vulnerability arises when we consider the multiple scales at which this variation occurs, be it temporal (from minute to annual) or spatial (from centimetres to kilometres). We studied populations of a single widely distributed butterfly species, *Chlosyne lacinia*, to examine the physiological, morphological, thermoregulatory and biophysical underpinnings of adaptation to tropical and temperate climates. Microclimatic and morphological data along with a biophysical model documented the importance of solar radiation in predicting butterfly body temperature. We also integrated the biophysics with a physiologically based insect fitness model to quantify the influence of solar radiation, morphology and behaviour on warming impact projections. While warming is projected to have some detrimental impacts on tropical ectotherms, fitness impacts in this study are not as negative as models that assume body and air temperature equivalence would suggest. We additionally show that behavioural thermoregulation can diminish direct warming impacts, though indirect thermoregulatory consequences could further complicate predictions. With these results, at multiple spatial and temporal scales, we show the importance of biophysics and behaviour for studying biodiversity consequences of global climate change, and stress that tropical climate change impacts are likely to be context-dependent.

## 1. Introduction

While it has been suggested for decades that tropical species may have narrow thermal tolerance ranges relative to temperate species due to lower seasonality in the tropics [1], only in recent years has this pattern been demonstrated and recognized as important for understanding global change [2–4]. Specifically, while changes in temperature in future climates are projected to be smaller in the tropics than in temperate or polar regions [5], impacts of warming could be as great or greater for tropical species compared with species at higher latitudes [6–9]. However, there is a great deal of uncertainty in how warming might impact species globally as multiple evolutionary processes shape climatic adaption, including physiology, ecology and genetic diversity [10–13].

Intra-annual variation in temperature (i.e. seasonality) can have important implications for thermal tolerance, but thermal variation over other time scales and variation over spatial gradients also affect climate change vulnerability [2,14,15]. Habitat and behavioural factors can additionally influence a population's capacity to respond to climatic change at a regional, landscape or microclimatic scale [16–20]. For example, Huey *et al.* [21] showed that tropical forest lizards living in relatively homogeneous shaded habitats may be highly vulnerable to warming, while Logan *et al.* [22], using finer-spatial-scale thermal data, argued that forest lizards might not be so vulnerable due to high (and underestimated) spatial variance in temperature. Behavioural thermoregulation (e.g. shade-seeking) can also affect a species response to climate change [23–26]. Species interactions are critical such that extinction or distribution change of one species could result in the extinction of other members of that species interaction network [27].

Even before we are able to incorporate these additional factors, we must first understand the biophysical system and physiological ecology of a given species to effectively project how a regional warming trend is likely to affect it [28,29]. For example, Janzen's [1] hypothesis that lower intra-annual variation in temperature experienced by tropical organisms results in lower tolerance breadth has a largely untested, but critical, assumption that air temperature is equivalent to body temperature. If such variation is *not* equivalent (e.g. if body temperatures are largely overlapping between temperate and tropical species, in contrast to air temperature, as demonstrated by Janzen [1]), then the predicted high vulnerability of tropical species to warming might be overstated [30]. Incorporating biophysics and understanding variation in body temperature (rather than simply air temperature) are therefore important but frequently neglected components of climate change impact research [13,23,31].

Through modelling and analysis of an array of data (e.g. microclimate, morphology), we characterized the biophysics and thermal ecology of tropical and temperate populations of a single geographically widespread butterfly species, *Chlosyne lacinia* (Lepidoptera: Nymphalidae). Specifically, we used a biophysical model to incorporate local microclimatic variables (air temperature, ground temperature, wind speed and solar radiation) for body temperature simulations of the organisms in each population and used that model to examine geographical variation in body temperature. We integrated the biophysical model with a large-scale insect fitness model that approximates thermal tolerance (i.e. thermal performance curves) based on seasonal thermal variation [6,15] to explore potential impacts of climate change on temperate and tropical insect populations. Using the integrated biophysical fitness model, we also examined the role of morphology and behavioural thermoregulation in structuring climate change responses across latitude.

## 2. Material and methods

### (a) Study sites and organism

We studied populations of *C. lacinia* in North and Central America. This species of the subfamily Nymphalinae was chosen due to its close relatedness to the well-studied checkerspot butterflies (e.g. *Euphydryas editha* and *Melitaea cinxia*) [32]. In addition, it is one of the most broadly distributed butterflies in the Americas, with populations ranging from Argentina to California and New Mexico. Where present, it tends to be locally abundant so that sufficient sample sizes could be found at each of the study sites.

What is known of *C. lacinia* natural history mostly comes from studies in Texas. Habitat requirements include open land, high food plant density and nectar sources, including a variety of host plants within Compositae [33]. Adults fly year round in the tropics, but only in the summer in North America. However, peak activity in Central America also typically occurs in the summer, usually beginning in July and lasting through November. Bonebrake *et al.* [34] explored the possibility that *C. lacinia* may actually represent multiple cryptic species, and found that while its evolutionary history is not straightforward, the phylogeny is consistent with the hypothesis that one single butterfly species is broadly distributed across temperate and tropical habitats.

We chose four main sites to conduct the study in 2007 and 2008: the Southwestern Research Station, Arizona (AZ; 31°53′ N, 109°13′ W, altitude 1700 m); Indio, California (CA; 33°43′ N, 116°12′ W, altitude –4 m); Santa Rosa National Park, Area de Conservacion Guanacaste, Costa Rica (CR; 10°48′ N, 85°36′ W, altitude 300 m); and Ahuachupan, El Salvador (ES; 13°59′ N, 89°11′ W, altitude 300 m). The ES and AZ sites underwent the most intensive sampling and serve as focal sites (see electronic supplementary material S1 for details on the sites and populations).

### (b) Biophysical model and microclimatic variation

We used a biophysical model to predict body temperatures of a butterfly with given morphology under a set of microclimatic conditions (for details, see [28,35–38]; electronic supplementary material S2 and S3). The biophysical model incorporates a simple behavioural thermoregulatory function. At cold temperatures, butterflies orient themselves to maximize solar radiation, whereas at high temperatures, butterflies will orient away from direct solar radiation to minimize exposure to lethal temperatures [39]. The model incorporates this into the solar radiative heating rate by reducing the fractional absorption of direct sunlight by a factor of 0.65 when in avoidance position (i.e. reduction in radiative flux equivalent to 65% of a basking individual) [38]. Thermoregulatory behaviour differs between *Chlosyne* (dorsal basker) and *Colias* (lateral basker) [40]. However, both butterflies maximize radiation by basking and orienting towards direct sunlight and minimize radiation by avoiding sunlight and orienting away from the sun.

For microclimatic inputs, we used a HOBO Micro Station Data Loggers (Onset Computers Corporation, MAN-H21-002) to record climatic data every minute. Four sensors were attached to the micro station: a silicon pyranometer smart sensor (S-LIB-M003: resolution 1.25 W m^−2^), a wind speed smart sensor (S-WSA-M003: resolution 0.38 m s^−1^, starting threshold 1 m s^−1^), and two 8-bit temperature smart sensors (S-TMA-M0XX: resolution 0.4°C). For morphological inputs, we collected individuals from each of the sites and measured thorax diameter, body length, forewing length, hindwing absorptivity and fur thickness (electronic supplementary material, S1).

### (c) Model validation

To validate the biophysical model, we measured body temperature directly with thermocouples. We inserted a fine-gauge thermocouple wire (Omega TFIR-003-50) attached to a hand-held thermocouple thermometer (Omega HH603A) into the thoraces of anaesthetized or freshly killed females. The female was then placed next to the microclimate station (in open sun) and in a dorsal basking position. We recorded body temperature of the female every minute during the validation procedure and then compared the observed body temperature with the body temperature predicted by the biophysical model. We used a similar procedure involving thermocouples and caged butterflies to roughly characterize thermal performance curves (flight probability versus body temperature) in Arizona and El Salvador (see electronic supplementary material S4 for more details).

### (d) Climate change

To determine temperate and tropical population responses to climate change and warming (changes in mean temperature annually), we integrated the biophysical model with a generalized climate change impact model based on variation in thermal performance curves for insect species (fitness versus temperature) across latitude [6,15]. The impact model, as originally derived by Deutsch *et al.* [6], relates the seasonality of surface air temperature to site-specific insect physiology measures (specifically, warming tolerance; critical thermal maximum minus the habitat temperature and thermal safety margin; optimum temperature minus the habitat temperature) of 38 insect species, and uses that relationship to estimate thermal performance curves for insects globally. Based on projections of changes in air temperature over the next century, the model can then project changes in insect fitness globally based on the thermal performance curves.

Here, we modify the global impact model of Deutsch *et al.* [6] by incorporating solar radiation and estimating *T*_b_ of *C. lacinia* based on the biophysical model presented such that we have an approximation of the impact model for the species throughout the Americas. We input solar radiation data from the NASA International Satellite Cloud Climatology Project (ISCCP) [41] into the biophysical model as *R_S_*. For this model integration exercise, we set ground temperature *T*_g_ equal to air temperature *T*_a_ and assumed no wind. Following Deutsch *et al.* [6], we used 1950–2000 Climate Research Unit (CRU) data [42] for present-day surface air temperature data, but instead of calculating seasonality directly, we entered these air temperature data into the biophysical model. We then set the morphological parameters to match the average female El Salvador *C. lacinia* (see electronic supplementary material S1 and table S1), assumed individuals were in basking posture and solved for *T*_b_ as described in the biophysical model above. This produced a climatology (global monthly time series) in *C. lacinia* body temperature (specifically basking *C. lacinia*) with which we estimated seasonality in *T*_b_. We extracted *T*_b_ seasonality at locations where insect physiology data were available from Deutsch *et al.* [6] and then regressed the seasonality to the physiological parameters (warming tolerance and thermal safety margin).

Based on the statistical relationship between global insect physiology and *C. lacinia* body temperature seasonality, we then examined the impact of projected changes in body temperature on *C. lacinia* fitness. We calculated body temperature changes by using an average of two A2 emissions scenario GFDL model projections [15], and inputting changes in air temperature and solar radiation into the biophysical model (see R script in electronic supplementary material S5) as in the statistical evaluation described previously. We used climate simulation data from 2070 to 2100 (air temperature and solar radiation) as the future climate and subtracted out the simulated baseline twentieth-century climate to calculate body temperature change. We then examined the effect of body temperature change on fitness for each point in the grid of interest, which for *C. lacinia* encompasses most of the Americas. We looked at the effect of morphology by using El Salvador and Arizona morphology (average female characteristics) to measure projected body temperature change, and we looked at the effect of thermoregulation by using an avoidance posture in the biophysical model for future butterflies (2070–2100) and looking at the projected fitness changes.

## 3. Results

### (a) Model validation, morphology and microclimate

**Figure 1.**
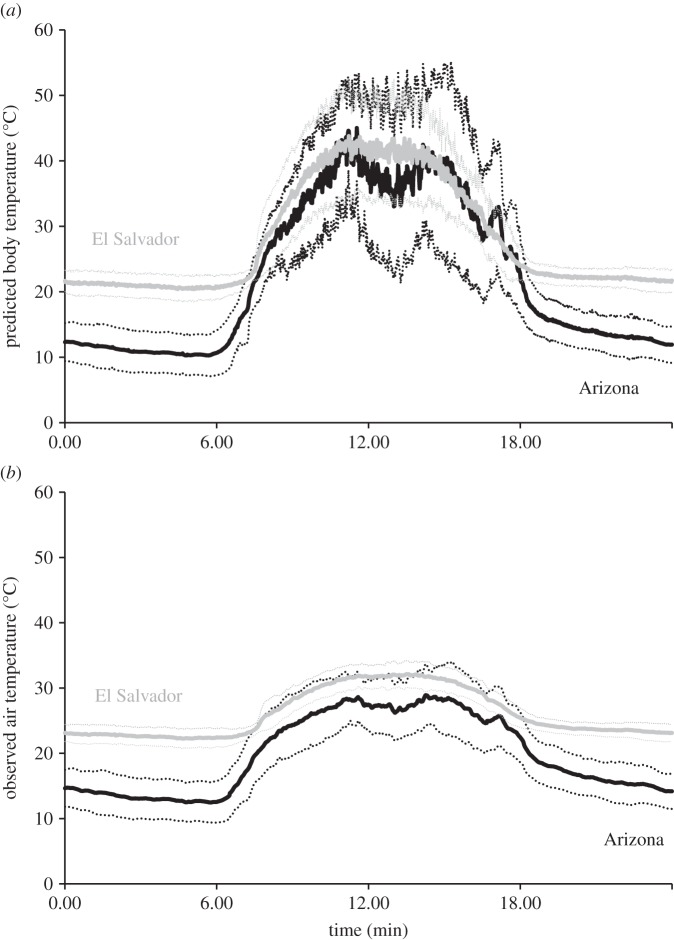
(*a*) Diurnal variation of *T*_b_ (mean, solid line; standard deviation, dotted lines) based on model for El Salvador and Arizona *C. lacinia*. Dip in *T*_b_ in the mid-afternoon hours in Arizona is a shading effect of a nearby tree. (*b*) The same for air temperature.

The biophysical model effectively predicted variation in *C. lacinia* body temperature (electronic supplementary material S4). Based on the biophysical model, while the body temperature of *C. lacinia* individuals is lower at night and during early morning hours, there is a great deal of overlap between El Salvador and Arizona in the late morning and afternoon hours (figure 1). Peak body temperatures (*T*_b_) for butterflies at both sites during late morning/early afternoon hours are predicted to be about 41°C, although using avoidance posturing butterflies can reduce peak *T*_b_ to between 35°C and 39°C. Diurnal variation (standard deviation of hourly means) in *T*_b_ (basking body temperature) was 8.4°C (s.d.) for El Salvador and 11.6°C for Arizona. Variation in air temperature (*T*_a_) was 3.7°C for El Salvador and 6.0°C for Arizona.

### (b) Impacts of climate change

**Figure 2.**
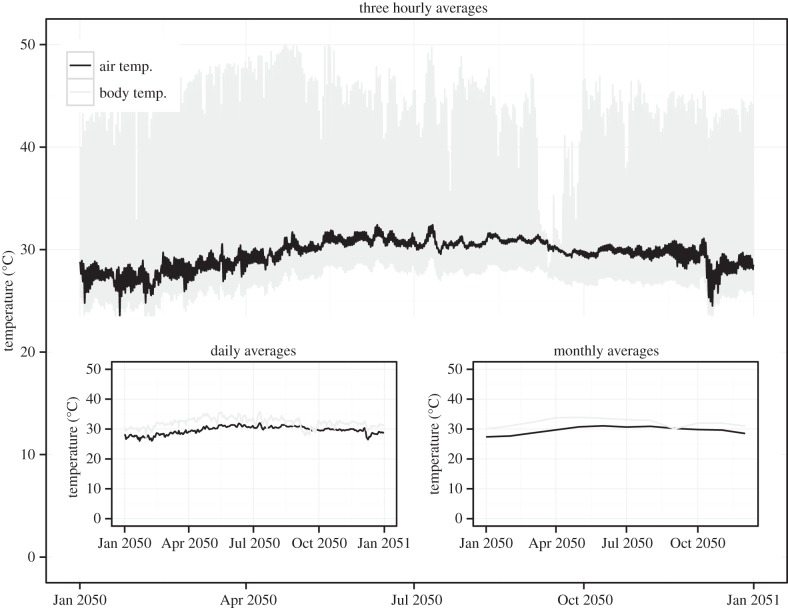
Air temperature variation (black) and *C. lacinia* avoidance body temperature variation (grey) based on solar radiation and air temperature projections (GFDL A2) at three-hourly intervals for the year 2050 (see electronic supplementary material S5 for details). Air and body temperature averaged over days (left inset) and months (right inset) also displayed.

Global modelling (versus microclimatic) of *C. lacinia* body temperature projected with a biophysical model incorporating solar radiation versus air temperature showed additional high variability in body temperature relative to air temperature, particularly at high temporal resolution (figure 2). The positive correlation between large-scale (1 km) variation in predicted *C. lacinia* body temperature, insect warming tolerance and thermal safety margins from available studies was significant (*R*^2^ = 0.43, *p* < 0.001 and *R*^2^ = 0.43, *p* < 0.001, respectively), but the relationship is not as strong as warming tolerance for insects and air temperature as analysed by Deutsch *et al.* [6]. Using this relationship (body temperature variation versus warming tolerance and thermal safety margin), we then estimated thermal performance curves for *C. lacinia* globally. In the absence of extensive *C. lacinia* thermal performance data, this is a necessarily imperfect association between multiple insect thermal performances and *C. lacinia* body temperature. However, the relationship provides a first approximation of how individual and thermoregulatory characteristics might affect the impacts of climate change on insect species.

**Figure 3.**
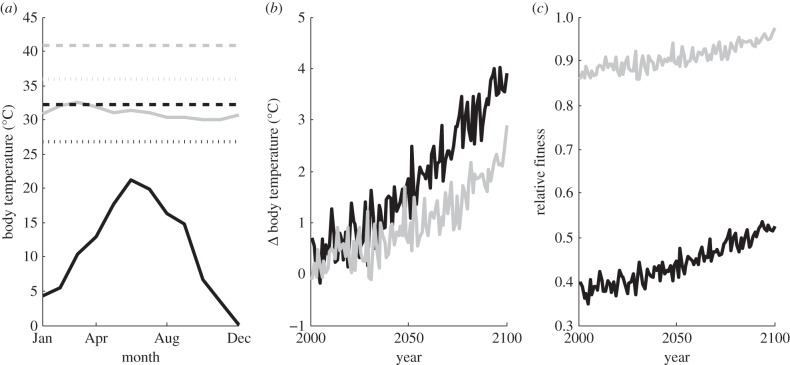
(*a*) Variation in monthly mean *C. lacinia* body temperature estimates based on observed surface air temperature (CRU) and solar radiation (ISCCP) for Arizona (black) and El Salvador (grey) with theoretically derived critical thermal maximum values or the end of the thermal performance curve where fitness declines to zero (dashed horizontal lines) and thermal optimum estimates (dotted horizontal lines). (*b*) Projected changes in *C. lacinia* body temperature based on projected changes in air temperature and solar radiation (GFDL). (*c*) Projected change in relative fitness given the estimated thermal performance curve and body temperature change.

**Figure 4.**
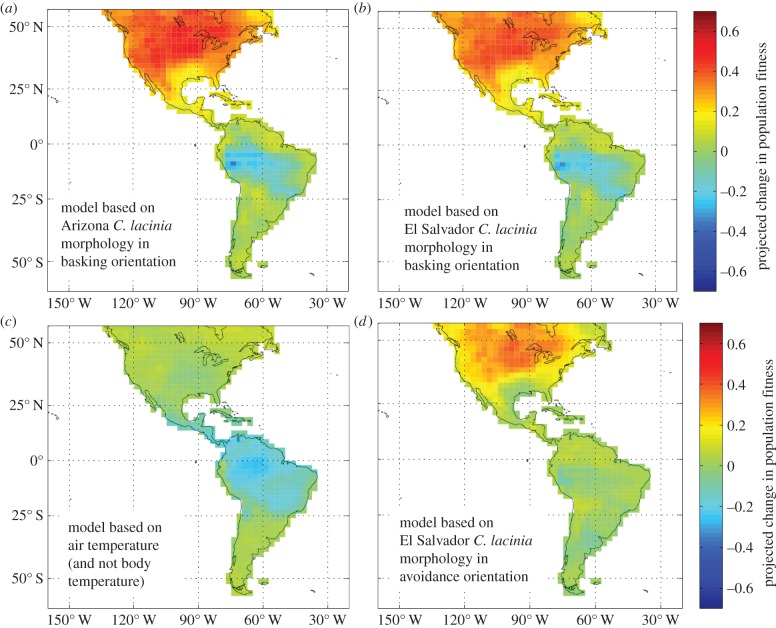
Climate change impacts (as measured by intrinsic population growth rates) on *C. lacinia* based on a relationship between insect physiology (intrinsic population growth rate as a function of temperature) and *C. lacinia* body temperature seasonality. Results are shown for *C. lacinia* morphologies typical of (*a*) temperate Arizona and (*b*) tropical El Salvador in basking orientation. (*c*) Fitness impacts based solely on air temperature variation and changes (as in Deutsch *et al.* [6]) and (*d*) a tropical (El Salvador) *C. lacinia* incorporating solar radiation and avoidance posture.

Using the climate projections and warming tolerance relationship, climate change is projected to increase both body temperatures and relative fitness in El Salvador and Arizona *C. lacinia* populations (figure 3). Across latitude generally, climate change is projected to differentially affect insect populations (figure 4). Regardless of morphology in *C. lacinia*, which has little effect on the results, some tropical populations are likely to experience negative fitness impacts (especially in South America) due to warming, while temperate populations (and some tropical populations) are expected to exhibit population growth (figure 4). However, reductions in the effects of solar radiation through thermoregulation (in this case, using avoidance posturing) can largely diminish this threat and erase much of the negative fitness impact for tropical insect populations (figure 4).

## 4. Discussion

Based on the morphological, biophysical and climate change model results, tropical ectotherm populations may be more vulnerable to climate change than temperate populations, but impacts will depend heavily on thermoregulatory behaviour, habitat and regional contexts. These results generally corroborate previous studies based on air temperature changes that have demonstrated significant climate change threats to tropical organisms [6,15]. On the other hand, the results also show that heat avoidance behavioural responses can alter climate change impact projections and the negative consequences for tropical organisms can be diminished. Sunday *et al.* [20] suggested in a recent analysis of multiple ectotherm taxa (using a lizard biophysics model) that thermoregulatory behaviour will be a *necessity* for organisms to avoid warming impacts in the future. Yet there are fitness costs to behavioural thermoregulation (e.g. a butterfly cannot fly or reproduce when in heat avoidance posture). That tropical ectotherms are likely to be threatened by future climate change therefore remains a troubling prospect given these results together.

Though we have long known that organismal body temperature does not vary directly and linearly with changes in air temperature [27], the importance of including body temperature at large spatial scales for global climate change impact models is relatively new [22,28,43]. Here, we established that air temperature variation was not a good surrogate for body temperature variation in *C. lacinia*. Solar influences on body temperatures are particularly likely to have critical effects on organismal physiology [44,45]. Other regional climatic factors not included in this model such as surface temperatures (ground, plants, etc.), wind and precipitation are furthermore likely to affect impacts of climate [27,46,47], emphasizing the need for further research in biophysical components of global change ecology models [29].

Similarly, using biophysical and solar radiative forcings allows for more detailed behavioural analyses critical for understanding of climate change impacts [48]. Our results suggest that many tropical insects (and other organisms) of open habitats may be able to escape many of the direct consequences of warming by avoiding sun behaviourally through shade-seeking, orienting away from the sun or otherwise minimizing solar radiation. However, adult butterflies also have high behavioural flexibility relative to other life stages (eggs, larvae and pupae). Warming impacts may therefore not be avoidable for much of an organism's life cycle [23,45], though individuals can further respond to stress through diapause and plasticity, potentially [49]. The incorporation of habitat and landscape features can further provide for more complex behavioural responses to warming impacts [21,22].

Thermal variation is exhibited at multiple spatial and temporal scales. Temporally, recent studies have shown the importance of examining diurnal, seasonal and inter-annual time scales for understanding warming impacts across latitude [2,6,50,51]. In addition to altering performance curves [49], temporal variation can be critical for species in buffering climate change impacts, especially for species with limited behavioural thermoregulation capacity [52]. As shown in this study (figures 1 and 2), the variation in air temperature at different scales (minutes, hours, years) may not be entirely sufficient to characterize the thermal environment given the high variation in body temperature at these scales. Spatial thermal heterogeneity can similarly complicate warming predictions [15,26,43,53]. High-resolution spatial and temporal data will probably therefore be key in understanding larger-scale global change patterns [54].

Biophysical and mechanistic studies have significant predictive advantages in ecological climate warming models but also have the limitations that they are data-intensive and often case-specific [55]. Worse yet, even the most detailed biophysical models have limitations, such as lacking dynamic thermal profiles as a function of height [56]. Each of these concerns applies in this study because we include microclimate, thermal performance and morphological data for one species across latitude (*C. lacinia*), and yet even these data are incomplete (e.g. biophysics of juvenile stages and latitudinal variation in activity times in the field are not considered in this model). However, despite these limitations, the results presented here suggest that models that ignore biophysical and physiological factors do so at the risk of misrepresenting realistic thermal variation in the environment.

Starting at small temporal (e.g. minute) and spatial (e.g. metre) scales, we were able to identify the climatic parameters that dictate organismal experience of body temperature. Based on those results, we integrated the small-scale observations with a macroclimatic model that examined climate change effects at large temporal (century) and spatial (kilometre) scales. Future research will do well to manage the multiple scales at which climatic variation and adaptation are operating to achieve the goal of effectively assessing climate change vulnerability of species globally, and tropical species in particular.

## Supplementary Material

ESM
