# Supplementary material for: From global change to a butterfly flapping: biophysics and behaviour affect tropical climate change impacts
Source: Proc Biol Sci. 2014 Oct 22;281(1793):20141264. doi: 10.1098/rspb.2014.1264 (PMC4173678; doi:10.1098/rspb.2014.1264)
Supplement: ESM [file rspb20141264supp1.pdf]

Electronic Supplementary Material

From global change to a butterfly flapping: biophysics and behaviour affect tropical climate change impacts

Electronic supplementary material S1: *Chlosyne lacinia* morphology across latitude

Site Description

Collections in Indio CA were done along roadsides at the city's edge near host-plant *Helianthus* clusters. In AZ, the main study site was at the Southwestern Research Station. In 2008 however, we collected *C. lacinia* larvae throughout Cochise County ranging from and into the western edge of Hidalgo County in New Mexico. The habitats occupied by *C. lacinia* ranged from alpine desert ecosystems to lowland abandoned agricultural land. The larvae were reared in laboratory facilities and the adults from those broods were used for data collection. Study in Ahuachupan El Salvador was centralized on a working-landscape butterfly farm situated on the Western border of the El Imposible National Park rainforest. Butterflies were typically found flying near host-plant *Verbesina* species and flowering nectar sources. In Santa Rosa National Park, CR we collected individuals in clearings surrounded by seasonally dry tropical forest.

Morphology measurement and statistics

For measurements of morphology, we collected individuals from each of the sites. We measured thorax diameter, body length and forewing length for each specimen using an electronic digital caliper. For fur thickness, we took digital photos of each specimen under a microscope (Leica S6D) with a micro ruler (with .1 mm increments) for scale. We analyzed the photos in ImageJ (http://rsb.info.nih.gov/ij) measuring the length of five individual setae lengths and taking the average of all for the estimate of fur thickness. For absorptivity (α), we used a spectroreflectometer (FieldSpec Pro) to measure wing reflectance and we subtracted from 1 the value of reflectance at the 650 wavelength (Stamberger 2006). *C. lacinia* individuals bask dorsally with wings spread and so we focused measurement on the dorsal median area of the wing (see Kingsolver 1985 for more discussion of thermoregulatory behavior in Lepidoptera). To examine the differences in morphology between populations and sexes, we ran a two-way ANOVA (with sex and population as factors) for each of the five characteristics of interest.

Both sexes and populations differed morphologically (Tables S1 and S2). Thorax size was generally larger in temperate populations and males generally had smaller thorax sizes than females. Body length was generally longer in tropical populations with no differences between sexes. Forewing length had little difference between sites or populations but males generally had shorter wings. Fur thickness was slightly larger in males and exhibited significant variation across populations and distributions. Finally, wing absorptivity did not vary significantly with sex or population.

Table S1. Mean and standard deviation of morphological *Chlosyne lacinia* features by population and sex.

Location	Sex	Thorax Diameter (mm)	Body Length (mm)	Forewing Length (mm)	Fur Thickness (mm)	Wing Absorptivity (%)

Indio, CA	Female	2.7±0.1	12.8±0.9	21.5±0.7	0.35±0.09	94±2.1
	Male	2.6±0.3	12.6±0.7	19.5±1.5	0.42±0.12	93±3.3
SWRS, AZ	Female	3.0±0.3	13.9±1.2	23.9±1.8	0.57±0.14	95±2.0
	Male	2.7±0.3	13.6±1.2	21.1±1.7	0.58±0.14	95±1.7
Ahuachupan, El Salvador	Female	2.5±0.2	14.1±0.9	24.0±1.8	0.44±0.10	95±1.9
	Male	2.4±0.3	14.1±1.0	22.5±1.5	0.51±0.10	95±1.4
Guanacaste, Costa Rica	Female	2.5±0.2	13.6±1.4	23.6±0.9	0.43±0.10	94±2.4
	Male	2.6±0.2	14.2±1.5	23.7±1.7	0.48±0.06	96±1.0

Table S2. Nested analysis of variance examining the effect of sex and distribution (temperate vs. tropical) on morphology (with population nested in distribution).

Morphological Feature	Scale	df	Mean Square Error	F-ratio	p
Thorax Diameter	Distribution	2	14118	14.11	<0.001
	Population (Distribution)	2	1361	1.36	0.26
	Sex	2	5124	5.12	0.006
	Residuals	235	1001		
Body Length	Distribution	2	19935	7.70	<0.001
	Population (Distribution)	2	13879	5.36	<0.001
	Sex	2	2353	0.91	0.40
	Residuals	235	2589		
Forewing Length	Distribution	2	4950	1.80	0.17
	Population (Distribution)	2	8079	2.94	0.05
	Sex	2	54112	19.70	<0.001
	Residuals	235	2747		
Fur Thickness	Distribution	1	0.207	16.11	<0.001
	Population (Distribution)	2	0.206	16.04	<0.001
	Sex	2	0.059	4.60	0.01
	Residuals	219	0.013		
Wing Absorptivity	Distribution	1	0.0001	0.36	0.55
	Population (Distribution)	2	0.0014	4.73	0.01
	Sex	2	0.0002	0.77	0.47
	Residuals	108	0.0003		

Electronic supplementary material S2: Biophysical model details

The energy balance of the organism is determined by three heating and cooling rates (all in watts): the solar radiative heating rate (Q_s), the thermal radiative heating (or cooling) rate (Q_t), and convection (Q_c). These rates are determined by microclimatic variables and morphological characteristics of the butterfly, according to the following equations:

$$Q_t = A(\sigma T_b^4 - R_L)$$
$$Q_s = A_S(f_{dir} - f_{ind})\alpha R_S$$
$$Q_c = Ah_t(T_b - T_a)$$

where T_b and T_a are the body and ambient air temperatures, R_L and R_S are the long-wave (infrared) and short-wave (solar) radiative fluxes (both in W/m^2), and A and A_S are the respective areas over which those fluxes are absorbed. The butterfly is assumed to emit radiation as a black-body at a rate of σT_b^4 (in W/m^2), where σ is the Stefan-Boltzmann constant, and to exchange heat with the ambient air in proportion to the body-air temperature difference with a wind-dependent rate coefficient, h_t. The solar heating depends on absorptivity (α), and the fractional absorption of direct and indirect (reflected) sunlight, via the factors f_{dir}=0.92 and f_{ind}=0.38, respectively (Kingsolver 1983). The long-wave radiative flux from the environment is estimated from temperature data as:

$$R_L = 0.5\sigma((1.2T_a - 348)^4 + T_g^4)$$

which accounts for clear-sky emissions from both the atmosphere and ground, according to their respective temperatures, T_a and T_g (Gates 1980). The area over which convective and thermal energy is exchanged depends on morphological characteristics of the butterfly: body length (L), thorax radius (r_{th}), and fur thickness (Th_f : length of ventral thoracic setae). h_t is calculated based on Th_f and r_{th} (see Kingsolver and Moffat 1982 and electronic supplementary material S1). Here we set $A_S = 2(r_{th} + Th_f)L$, the cross-sectional area, whereas $A = 2(r_{th} + Th_f)L\pi 0.8$ approximates the total surface area.

In a steady-state with no net energy flow, the heat gained by short wave radiation (Q_s) equals the heat lost by convection (Q_c) and long wave radiation (Q_t).

$$Q_s = Q_t + Q_c$$

Because these energy exchanges in turn depend on body temperature, a steady state can only be achieved at a particular body temperature, which can be solved numerically under specified biophysical and climatic conditions (see R script in electronic supplementary material S3).

The microclimate sensors recorded solar radiation (in W/m^2 but converted into mW/cm^2 for the model), wind speed (in *m/s* but converted into *cm/s*), air temperature (in °C), and ground temperature (in °C) respectively. The air temperature sensor was equipped with a solar radiation shield (RS3) and placed approximately half a meter off the ground. This represented *C. lacinia* basking conditions, because a majority of the butterflies bask on shrubs at about that height. The HOBO Micro Station Data Loggers were set in locations where *C. lacinia* flight was observed

and in locations relatively free of surrounding high vegetation which could confound measurements of light intensity. HOBO Micro Station Data Loggers were set up in 2008 in the focal populations for a total of 322 hours between August 10 2008 and September 18 2008 (19,331 data points) at the Southwestern Research Station in Arizona and 768 hours between October 20 2008 and November 23 2008 (46,080 data points) in El Salvador. Days on which equipment malfunctions occurred (e.g., ground temperature sensors were blown out of the ground) were removed from the analysis.

References

Gates D. M. 1980 Biophysical ecology. New York: Springer-Verlag.

Kingsolver J. G., Moffat R. J. 1982 Thermoregulation and the determinants of heat transfer in Colias butterflies. Oecologia 53, 27–33.

Kingsolver J. G. 1983 Thermoregulation and flight in Colias butterflies: Elevational patterns and mechanistic limitations. Ecology 64, 534–545.

Kingsolver J. G. 1985 Butterfly thermoregulation: organismic mechanisms and population consequences. J. Res. Lepidoptera 24, 1–20.

Stamberger J. A. 2006 Adaptation to temporal scales of heterogeneity in the thermal environment. Stanford University, Dissertation.

Electronic supplementary material S3: Heat transfer model using microclimate data

```
#R script (version S3) for a heat transfer model to calculate as the output
butterfly body temperature (degrees C) in basking and heat avoidance postures
by Bonebrake et al, using microclimate data. The model assumes steady state
energy flux and takes into account radiative and convective heat transfer
(conduction through insect feet contact to ground or vegetation is considered
negligible).

#This model was originally developed for Colias butterflies by Kingsolver
(Kingsolver, J. G., & Watt, W. B. (1984). Mechanistic constraints and
optimality models: thermoregulatory strategies in Colias butterflies.
Ecology, 1835-1839.), then was modified by Watt (Watt, W. B. (1992). Eggs,
enzymes, and evolution: natural genetic variants change insect fecundity.
Proceedings of the National Academy of Sciences, 89(22), 10608-10612.), then
was transliterated to MatLab by Stamberger (2006). The model has been adapted
for R and Chlosyne butterflies by Bonebrake et al. Refer to Bonebrake et al.
(2014) for equation details.

#The program should be cited as Bonebrake, T.C., Boggs, C.L., Stamberger,
J.A., Deutsch, C.A., Ehrlich, P.R. From global change to a butterfly
flapping: biophysics and behaviour affect tropical climate change impacts. R
script version S3.

#This version (S3) uses inputs from microclimate measures (air temperature,
ground temperature, solar radiation and windspeed). Version S5 incorporates
inputs from regional climate predictions of air temperature and solar
radiation, ignoring impacts of ground temperature and wind speed.

#Inputs to this version include: microclimate data (air temp, ground temp,
solar radiation and wind speed) and morphology (body length, "fur" length and
absorptivity).

#This model has been validated for a lateral basking butterfly Colias meadii
(Kingsolver and Watt 1984) and in Bonebrake et al. for a dorsal basking
butterfly, Chlosyne lacinia. This same model could potentially apply to a
wider range of small ectotherms.

#R Packages
# Requires R statistical package “pracma”  pracma: Practical Numerical Math
Functions: Functions from numerical analysis and linear algebra, numerical
optimization, differential equations, plus some special functions. Uses
Matlab function names where appropriate to simplify porting.
# code to install pracma: install.packages("pracma", repos="http://R-Forge.R-
project.org")

#Inputs: Microclimate data
#Microclimate data is contained in a tab delimited .txt file structured as
follows:Column 1=Year, Column 2=Julian Day, Column 3=Hour (0-23), Column
4=Minute, Column 5=Air Temp (deg C), Column 6= wind speed (m/s), Column 7=
ground temp (deg C), Column 7= short wave radiation (cm/mW). For
demonstration purposes the microclimate data file here is called
“chlosyneinput.txt”, and has the following content:
#2008 289   0     0     22.657      0     25.56 0.06
#2008 289   0     1     22.657      0     25.56 0.06
#2008 289   0     2     22.657      0     25.56 0.06
```

```
#2008 289   0     3     22.609      0     25.56 0.06
#2008 289   0     4     22.609      0     25.56 0.06
#2008 289   0     5     22.561      0     25.56 0.06
#2008 289   0     6     22.561      0     25.56 0.06
#2008 289   0     7     22.537      0     25.56 0.06
#2008 289   0     8     22.513      0     25.56 0.06
#2008 289   0     9     22.513      0     25.56 0.06
#2008 289   0     10    22.513      0     25.56 0.06
#2008 289   0     11    22.465      0     25.56 0.06
#2008 289   0     12    22.465      0     25.56 0.06

rm(list=ls(all=TRUE))
#Define variable A as the matrix of the microclimate data in the input file
A=as.matrix(read.table('chlosyneinput.txt'))
#Alength is the number of rows in the microclimate data file, used at the end
of the program for iteratively calculating body temperature for each row of
the microclimate data file
Alength <-length(A[,5])
#Morphological inputs
#For demonstration purposes, inputs below are provided below for a female El
Salvador Chlosyne specimen (SpecimenID), with a body length (LEN in cm) of
1.375cm, thoracic radius (RO in cm) of 0.96cm, absorptivity value (ALPHA) of
0.96, "fur" length (FUR in cm) of 0.0548cm, and an elevation of 1km.
LEN <- 1.375
RO <- .243
ALPHA <- .96
FUR <- 0.0548
ELEV<- 1

#Thermal constants
SIGMA <- 5.67E-9
KEFF <- 1.3

#Surface areas for convection (ARADCONV) direct radiation (ADIRRAD)
D<-2*(RO+FUR)
ARADCONV <-(D*LEN*pi)*.80
ADIRRAD <- D*LEN

#Free and Forced Convection (NEWCOOLK)
   PR <- 101325.0*((1-(0.0226*ELEV))^5.255)
   AIRDEN <- PR /(287.04*(A[,5]+273))
   DYNVISC <- 0.183*((416.2/(393.2+A[,5]*((A[,5]+273)/296)^1.5)))
   KINVISC <- DYNVISC/AIRDEN
   THCONDAIR <- 0.2425 + ((A[,5])*7.038E-4)
   REYNOLDS <- (D*A[,6])/KINVISC
   NUSSELT <- 0.59*(REYNOLDS^0.5)
   invisible(ifelse(NUSSELT < 2.3, NUSSELT <- 2.3, NUSSELT <- NUSSELT))
      HC<-(THCONDAIR*NUSSELT)/D
   invisible(ifelse(FUR<0,HT<-HC,HT<-
(KEFF/(RO*log((RO+FUR)/RO))*HC)/(KEFF/(RO*log((RO+FUR)/RO))+HC)))
      NEWCOOLK<-ARADCONV*HT

#Long-wave radiation from the sky(SKYRAD) and ground (GRDRAD)
  TSKY <- 273.0 +(A[,5]-21.0)+(0.2*A[,5]);
  SKYRAD <- TSKY^4;
  GRDRAD <-(A[,7]+273)^4;
```

```
#Short wave radiation in basking orientation (QDIRBASK) heat avoidance
orientation (QDIRAVOID), and heat coming into the body from solar radiation
that is reflected off the ground/ vegetation (QREST).
QDIRBASK <-(0.92*ADIRRAD*1.0*ALPHA*A[,8])
QDIRAVOID <-(0.92*ADIRRAD*0.65*ALPHA*A[,8])
QREST <- (0.38*ADIRRAD*ALPHA*A[,8])

#Solve for body temperature in basking orientation
require(pracma)
baskvars <- data.frame(QDIRBASK,QREST,NEWCOOLK,A[,5],SKYRAD,GRDRAD)
baskvarsm <- data.matrix(baskvars)

#Create a zero length vector called fbasksol, which will store calculated and
output values of body temperature in basking orientation for each line of
microlimate data
fbasksol <- numeric(Alength)
#Calculate body temperature in basking orientation for each line of
microclimate data, to be stored in fbasksol
for (i in 1:Alength)
 {
fbasksolve <- fzero(function(p) baskvarsm[i,1] + baskvarsm[i,2] -
(baskvarsm[i,3]*(p - baskvarsm[i,4]))-(((ARADCONV/2)*SIGMA*(((p+273)^4)-
baskvarsm[i,5]))+((ARADCONV/2)*SIGMA*(((p+273)^4)-baskvarsm[i,6]))),c(0,80))
fbasksol[i] <-fbasksolve$x
 }

#Solve for body temperature in avoidance orientation
avoidvars <- data.frame(QDIRAVOID,QREST,NEWCOOLK,A[,5],SKYRAD,GRDRAD)
avoidvarsm <- data.matrix(avoidvars)

#Create a zero length vector called favoidsol, which will store calculated
and output values of body temperature in heat avoidance orientation for each
line of microlimate data
favoidsol <- numeric(Alength)
#Calculate body temperature in heat avoidance orientation for each line of
microclimate data and store in favoidsol
for (i in 1:Alength)
 {
favoidsolve <- fzero(function(p) avoidvarsm[i,1] + avoidvarsm[i,2] -
(avoidvarsm[i,3]*(p - avoidvarsm[i,4]))-(((ARADCONV/2)*SIGMA*(((p+273)^4)-
avoidvarsm[i,5]))+((ARADCONV/2)*SIGMA*(((p+273)^4)-
avoidvarsm[i,6]))),c(0,80))
favoidsol[i] <-favoidsolve$x
 }
#Expected body temperatures (outputs) given chlosyneinput.txt input file:
#fbasksol 20.35650 20.35650 20.35650 20.31697 20.31697 20.27744 20.27744
20.25767 20.23791 20.23791 20.23791 20.19838 20.19838
#favoidsol 20.35169 20.35169 20.35169 20.31216 20.31216 20.27263 20.27263
20.25286 20.23310 20.23310 20.23310 20.19357 20.19357

#Plot of avoidance and basking body temperatures over the time series
plot(fbasksol,type="l",col="red")
lines(favoidsol,col="black")

#Writes output to a text file
bodytemps <- cbind(A, fbasksol,favoidsol)
write.table(bodytemps, file="output.txt", sep="\t",append=F)
```

Electronic supplementary material S4: *Chlosyne lacinia* thermocouple measurements for biophysical model validation and a preliminary investigation of flight performance and temperature

Biophysical model validation

Air temperature was not a good surrogate for butterfly body temperature (Fig. S1). Direct sun exposure caused significant rises in T_b but had little effect on T_a such that mean T_a during the model validation morning trials was 29.4±2.2 (mean±standard deviation) but mean observed T_b was 38.3±4.7 and modelled T_b was 39.2±5.9. The model T_b does appear to overpredict observed T_b at times (i.e., modelled temperatures are biased high and above observed) particularly during late morning/ afternoon hours. Wind is also an important determinant of body temperature for insects and *C. lacinia*, but the low resolution of our wind speed sensor (breezes and wind speeds below 1 *m/s* were not logged) likely underestimated its importance. This could also be the reason our modeled body temperatures frequently overpredicted the observed *C. lacinia* body temperatures.

Live medium-sized butterflies (such as *C. lacinia*) generate little heat through behaviours such as wing flapping and their body temperatures can therefore be approximated by measuring thermocoupled butterflies that are paralyzed, anesthetized or recently deceased (Kingsolver 1983). A butterfly's orientation to the sun however can affect its body temperature significantly (Kingsolver 1983) and so this is the bevioural thermoregulatory response (basking vs. heat avoidance) that we focus on in the model.

Flight response to thermal variation in Arizona and El Salvador

We used a thermocouple technique similar to the biophysical model validation in order to measure flight propensity. In ES we took individuals with inserted thermocouples and placed them in open habitat with caged individuals. We then recorded the body temperature of the thermocoupled individual as well as whether or not the individual was flying (if alive) and whether or not the caged individuals were flying. We conducted this experiment for a total of 31 hours. In AZ we took 30 butterflies and placed them in an environmental chamber (Percival). We placed in the chamber three additional butterflies with thermocouples attached. We started the experiment at 15°C and ended at 45°C. For each degree change we noted the body temperatures of the thermocoupled butterflies to determine equilibration; with negligible effects of wind or solar radiation butterfly body temperature equilibrated with air temperature after about five minutes. We also noted how many butterflies were flying for each degree. The different methodologies at the different sites were due to necessity. Environmental chambers were not available at the ES site (among many other reasons, there was no electricity).

Butterflies in AZ begin flying at much lower body temperatures than ES butterflies (Figure S2). The lower limit of flight activity range for El Salvador *C. lacinia* was 27°C while Arizona *C. lacinia* began flying at 21°C. The upper limit for flight activity in Arizona was 43°C. Flight activity did not decrease at high temperatures for *C. lacinia* in El Salvador but this is likely due to the different techniques used for flight activity assessment (lab vs. field collected data). For example, 49 butterflies died during the experiment in El Salvador and the average temperature reading of the thermocouple-implanted butterfly at the time of lethality was 41.6°C. Thus, the thermal tolerance upper limit for *C. lacinia* in El Salvador is at least roughly comparable to the upper limit in Arizona.

Figure S1. Running means of observed air temperatures, modeled and observed female *C. lacinia* body temperatures in El Salvador over four mornings of data collection on a minute by minute basis.

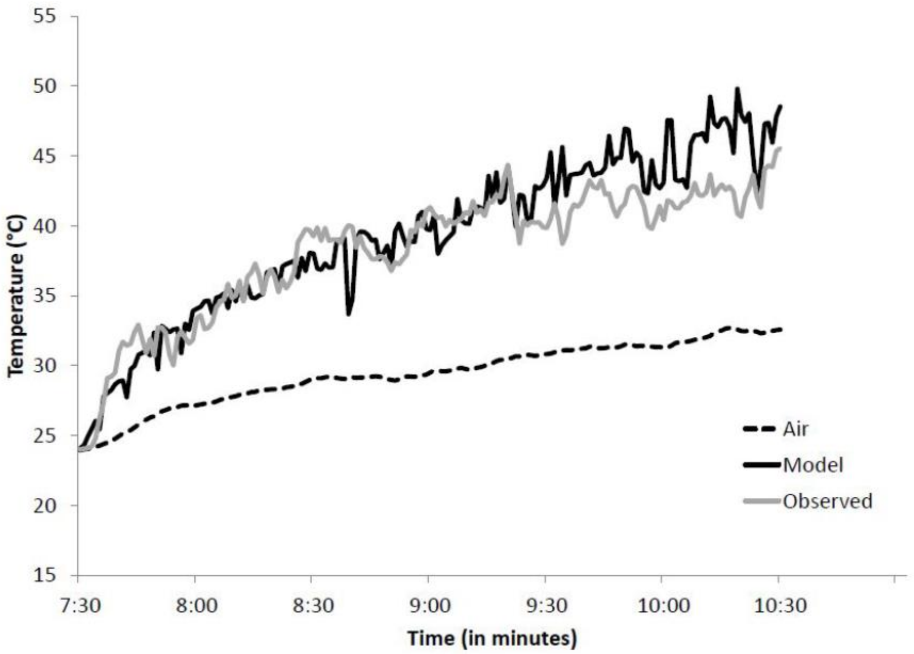

Figure S2. Body temperature and its relationship with flight probability are displayed for *C. lacinia* from El Salvador (blue line and dots) and Arizona (environmental chamber: gray line and dots, field: gray dashed line and Xs).

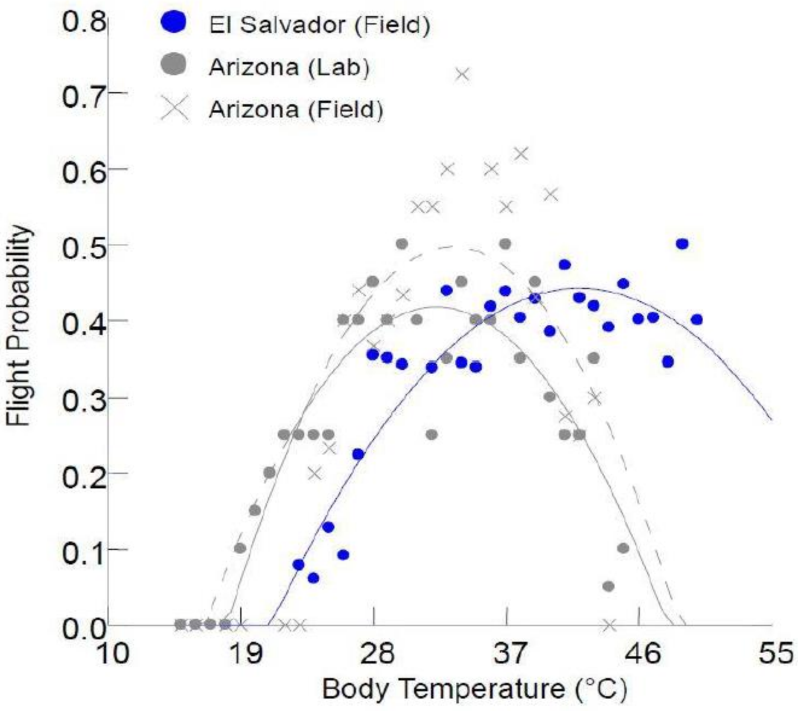

Electronic supplementary material S5: Heat transfer model using regional climate data

```
#R script (version S5) for a heat transfer model to calculate as the output
butterfly body temperature (degrees C) in basking and heat avoidance postures
by Bonebrake et al, given regional climatic inputs. The model assumes steady
state energy flux and takes into account radiative and convective heat
transfer (conduction through insect feet contact to ground or vegetation is
considered negligible).

#This model was originally developed for Colias butterflies by Kingsolver
(Kingsolver, J. G., & Watt, W. B. (1984). Mechanistic constraints and
optimality models: thermoregulatory strategies in Colias butterflies.
Ecology, 1835-1839.), then was modified by Watt (Watt, W. B. (1992). Eggs,
enzymes, and evolution: natural genetic variants change insect fecundity.
Proceedings of the National Academy of Sciences, 89(22), 10608-10612.), then
was transliterated to MatLab by Stamberger (2006). The model has been adapted
for R and Chlosyne butterflies by Bonebrake et al. Refer to Bonebrake et al.
(2014) for equation details.

#The program should be cited as Bonebrake, T.C., Boggs, C.L., Stamberger,
J.A., Deutsch, C.A., Ehrlich, P.R. From global change to a butterfly
flapping: biophysics and behaviour affect tropical climate change impacts. R
script S2.

#This version (S5) uses inputs from regional climate predictions/observations
of air temperature and solar radiation, ignoring impacts of ground
temperature and wind speed to predict butterfly body temperature. Version S3
predicts body temperature based on microclimate measures (air temperature,
ground temperature, solar radiation and windspeed) of environmental
variation.

#Inputs to this version include: macroclimate data (air temp and solar
radiation) and morphology (body length, "fur" length and absorptivity).

#R Packages
# Requires R statistical package “pracma”  pracma: Practical Numerical Math
Functions: Functions from numerical analysis and linear algebra, numerical
optimization, differential equations, plus some special functions. Uses
Matlab function names where appropriate to simplify porting.
# code to install pracma: install.packages("pracma", repos="http://R-Forge.R-
project.org")

#Inputs: Macroclimate data
#Macroclimate data can be downloaded from a variety of distributors including
Climate Research Unit (http://www.cru.uea.ac.uk/) and Intergovernmental Panel
on Climate Change (http://www.ipcc-data.org/). Data are typically in NetCDF
format. Code provided below in the comments show an example of how to extract
relevant air temperature and solar radiation data, once the RNetCDF R package
is installed (http://www.unidata.ucar.edu/software/netcdf/).
#require(RNetCDF)
#SolarData<-open.nc("SolerNetCDF.nc")
```

```
#TemperatureData<-open.nc("TemperatureNetCDF.nc")
#SolarVector <- var.get.nc(SolarData, "rsds", start=c(x,y,z), count=c(x,y,z))
#TemperatureVector <- var.get.nc(TemperatureData, "tas", start=c(x,y,z),
count=c(x,y,z))

rm(list=ls(all=TRUE))

# For the purposes of testing this code, example temperature and solar
radiation vectors are provided.
TemperatureVector <- c(300.12,300.54,301.42,303.23,300.34)
SolarVector <- c(1,10,400,255,200)

#Input air temperature (TemperatureVector) and solar radiation (SolarVector)
data from climate projection data
#Units should be converted into degrees C (for temp) and mW/cm (for solar)
Temp <- (TemperatureVector-272.15)
SolarCM <- SolarVector/10

#Vlength is the number of rows in the climate vectors, used at the end of the
program for iteratively calculating body temperature for each row of the
climate vectors
Vlength <-length(Temp)

#Morphological inputs
#For demonstration purposes, inputs below are provided below for a female El
Salvador Chlosyne specimen (SpecimenID), with a body length (LEN in cm) of
1.375cm, thoracic radius (RO in cm) of 0.96cm, absorptivity value (ALPHA) of
0.96, "fur" length (FUR in cm) of 0.0548cm, and an elevation of 1km.
LEN <- 1.375
RO <- .243
ALPHA <- .96
FUR <- 0.0548
ELEV<- 1

#Thermal constants
SIGMA <- 5.67E-9
KEFF <- 1.3

#Surface areas for convection (ARADCONV) direct radiation (ADIRRAD)
D<-2*(RO+FUR)
ARADCONV <-(D*LEN*pi)*.80
ADIRRAD <- D*LEN

#Free and Forced Convection (NEWCOOLK)
  PR <- 101325.0*((1-(.0226*ELEV))^5.255)
   AIRDEN <- PR /(287.04*(Temp+273))
   DYNVISC <- 0.183*(416.2/(393.2+Temp*((Temp+273)/296)^1.5))
   KINVISC <- DYNVISC/AIRDEN
   THCONDAIR <- 0.2425 + ((Temp)*7.038E-4)
   REYNOLDS <- (D*0)/KINVISC
   NUSSELT <- 2.3
      HC<-(THCONDAIR*NUSSELT)/D
         Rtotal<-RO+FUR
         HF<-KEFF/(RO*log(Rtotal/RO))
         HT<-(HF*HC)/(HF+HC)
      NEWCOOLK<-ARADCONV*HT
```

```
#Long-wave radiation from the sky(SKYRAD) and ground (GRDRAD)
    TSKY <- 273.0 +(Temp-21.0)+(0.2*Temp);
    SKYRAD <- TSKY^4;
    GRDRAD <-(Temp+273)^4;


#Short wave radiation in basking orientation (QDIRBASK) heat avoidance
orientation (QDIRAVOID), and heat coming into the body from solar radiation
that is reflected off the ground/ vegetation (QREST).
QDIRBASK <-(0.92*ADIRRAD*1.0*ALPHA*SolarCM)
QDIRAVOID <-(0.92*ADIRRAD*0.65*ALPHA*SolarCM)
QREST <- ALPHA*SolarCM*0.38*ADIRRAD;

#Solve for body temperature in basking orientation
require(pracma)
baskvars <- data.frame(QDIRBASK,QREST,NEWCOOLK,Temp,SKYRAD,GRDRAD)
baskvarsm <- data.matrix(baskvars)

#Create a zero length vector called fbasksol, which will store calculated and
output values of body temperature in basking orientation for each line of
microlimate data
fbasksol <- numeric(Vlength)
#Calculate body temperature in basking orientation for each line of
microclimate data, to be stored in fbasksol
for (i in 1:Vlength)
 {
fbasksolve <- fzero(function(p) baskvarsm[i,1] + baskvarsm[i,2] -
(baskvarsm[i,3]*(p - baskvarsm[i,4]))-(((ARADCONV/2)*SIGMA*(((p+273)^4)-
baskvarsm[i,5]))+((ARADCONV/2)*SIGMA*(((p+273)^4)-baskvarsm[i,6]))),c(0,80))
fbasksol[i] <-fbasksolve$x
 }

#Solve for body temperature in avoidance orientation
avoidvars <- data.frame(QDIRAVOID,QREST,NEWCOOLK,Temp,SKYRAD,GRDRAD)
avoidvarsm <- data.matrix(avoidvars)

#Create a zero length vector called favoidsol, which will store calculated
and output values of body temperature in heat avoidance orientation for each
line of microlimate data
favoidsol <- numeric(Vlength)
#Calculate body temperature in heat avoidance orientation for each line of
microclimate data and store in favoidsol
for (i in 1:Vlength)
 {
favoidsolve <- fzero(function(p) avoidvarsm[i,1] + avoidvarsm[i,2] -
(avoidvarsm[i,3]*(p - avoidvarsm[i,4]))-(((ARADCONV/2)*SIGMA*(((p+273)^4)-
avoidvarsm[i,5]))+((ARADCONV/2)*SIGMA*(((p+273)^4)-
avoidvarsm[i,6]))),c(0,80))
favoidsol[i] <-favoidsolve$x
 }
#Expected body temperatures (outputs) given inputted climate vectors:
#fbasksol 20.35650 20.35650 20.35650 20.31697 20.31697 20.27744 20.27744
20.25767 20.23791 20.23791 20.23791 20.19838 20.19838
#favoidsol 20.35169 20.35169 20.35169 20.31216 20.31216 20.27263 20.27263
20.25286 20.23310 20.23310 20.23310 20.19357 20.19357

#Plot of avoidance and basking body temperatures over the time series
```

```
plot(fbasksol,type="l",col="red")
lines(favoidsol,col="black")

#Writes output to a text file
bodytemps <- cbind(Temp, SolarCM, fbasksol,favoidsol)
write.table(bodytemps, file="output.txt", sep="\t",append=F)
```
